# Cloud Based Surveys to Assess Patient Perceptions of Health Care: 1000 Respondents in 3 days for US $300

**DOI:** 10.2196/resprot.5772

**Published:** 2016-08-23

**Authors:** Jonah Bardos, Jenna Friedenthal, Jessica Spiegelman, Zev Williams

**Affiliations:** ^1^ Mount Sinai Medical Center Department of Obstetrics Gynecology and Reproductive Science Icahn School of Medicine New York, NY United States; ^2^ Program for Early and Recurrent Pregnancy Loss (PEARL) Department of Obstetrics and Gynecology and Women’s Health Albert Einstein College of Medicine New York, NY United States; ^3^ NYU Langone Medical Center Department of Obstetrics and Gynecology New York University New York, NY United States; ^4^ Program for Early and Recurrent Pregnancy Loss (PEARL) Department of Obstetrics and Gynecology and Women’s Health Albert Einstein College of Medicine Bronx, NY United States

**Keywords:** Mechanical Turk, MTurk, crowdsourcing, medical survey, cloud-based survey, health care perceptions

## Abstract

**Background:**

There are many challenges in conducting surveys of study participants, including cost, time, and ability to obtain quality and reproducible work. Cloudsourcing (an arrangement where a cloud provider is paid to carry out services that could be provided in-house) has the potential to provide vastly larger, less expensive, and more generalizable survey pools.

**Objective:**

The objective of this study is to evaluate, using Amazon's Mechanical Turk (MTurk), a cloud-based workforce to assess patients’ perspectives of health care.

**Methods:**

A national online survey posted to Amazon's MTurk consisted of 33 multiple choice and open-ended questions. Continuous attributes were compared using *t* tests.

**Results:**

We obtained 1084 responses for a total cost of US $298.10 in less than 3 days with 300 responses in under 6 hours. Of those, 44.74% (485/1084) were male and 54.80% (594/1084) female, representing 49 out of 50 states and aged 18 to 69 years.

**Conclusions:**

Amazon’s MTurk is a potentially useful survey method for attaining information regarding public opinions and/or knowledge with the distinct advantage of cost, speed, and a wide and relatively good representation of the general population, in a confidential setting for respondents.

## Introduction

Surveys are important research tools that allow researchers to obtain both quantitative and qualitative information from respondents that can assist health care providers and policy makers to improve education, direct research, and enhance patient care. However, obtaining generalizable and representative information in a timely and cost-efficient manner is a constant challenge in population-based surveys. Surveys have traditionally been administered in person, by phone or mail. Although this variety affords researchers greater freedom to collect data, administrative, economic and research design complications may arise, with inherent biases introduced. In-person surveys, for example, often require extensive training for interviewers; respondents may feel less comfortable answering in-person survey questions honestly, and it is challenging to survey a population over a large geographical region [1]. Phone surveys or “cold calling” can be time consuming, limited by language barriers, and biased toward respondents who have access to landlines and are willing to participate [2]. Mail surveys, meanwhile, require extensive amounts of time and money and often have a poor response rate, which can introduce bias and affect the validity of the study [3]. One study estimated the cost per completed validated mail survey at US $17 dollars [4]. Multiple recent studies have shown that with postal surveys it can take a minimum of 8 to 12 weeks to obtain the majority of responses with multiple reminders, with some surveys taking even longer [5-7]. A Cochrane review by Edwards et al [8] investigated strategies to improve response rates to questionnaires delivered via either postal mail or electronic mail and found that although certain strategies may improve response rates, the odds of responses reduced when the study included questions of a sensitive nature.

Over the last ten years, online surveys have become more frequently used [9]. Online surveys offer significant time and cost-effectiveness in their ability to reach a large, diverse audience worldwide, while eliminating the need to send study personnel to conduct interviews, make calls or print and manually distribute survey instruments. Further, the ability to fill out a survey online creates a greater sense of anonymity among participants, thus alleviating anxiety related to answering questions honestly [10,11]. Although online surveys represent a clear improvement in efficiency, they can be costly, typically US $1-3 per respondent [12].

Online crowdsourcing services such as Amazon’s Mechanical Turk (MTurk) are Internet-based marketplaces that connect businesses (termed “requesters”) with individuals interested in performing Internet tasks. Requesters post tasks and workers may then choose to complete any number of available tasks for the listed monetary compensation. Amazon’s MTurk, for example, accesses more than 500,000 workers from over 190 countries [13]. Concerns have been raised regarding the use of MTurk in human participant research. A study by Kuang et al [14] compared patient understanding and recognition of pictographs using MTurk versus traditional survey methods. They found that although MTurk may be complimentary to traditional surveys, the respondent population differed, with white, higher educated respondents over-represented in the MTurk sample as compared to respondents tested with in-person surveys. However, subsequent studies suggested that MTurk participants are more demographically diverse than the standard Internet population, and the data obtained are at least as reliable as those obtained via traditional methods [15]. In addition, concerns have been raised regarding the quality of work produced by MTurk participants, namely that the low median wage of MTurk workers may affect worker motivation, and ultimately, may affect the quality of work produced [16]. However, MTurk has been validated as a tool for conducting behavioral research several times over, including determining the effect of medical guidelines on behavior [17-20].

Our previous work looked at public perceptions of miscarriage and found that there are widespread misperceptions regarding the frequency and causes of miscarriage [21]. It found that patients who have had miscarriages frequently feel guilty, ashamed, and alone and suggested that revelations from friends and celebrities regarding their own losses can help assuage those feelings.

Few studies have assessed patient experiences and satisfaction with their reproductive health care. Prior surveys were in-person interviews or questionnaires, which introduced the possibility of responder biases. Such surveys, particularly when questioning respondents about highly sensitive health information, introduce responder and non-responder bias, respectively [22,23]. While some studies utilized MTurk to recruit patients to another survey site, none have used MTurk as their survey engine. To our knowledge, our previous study [21] represented the first use of a crowdsourcing service to obtain information regarding respondent attitudes, perceptions, and understanding of health issues. Here, we report on sampling and data acquisition using the crowdsourced online survey.

## Methods

### Setup and User Interface

We used a cloud-based, medical knowledge voluntary closed survey regarding public perceptions of miscarriage via Amazon’s Web service MTurk (Multimedia Appendix 1). MTurk is an online labor market in which “requesters” have access to recruit a large number of “workers” to complete tasks. Typical tasks include transcribing audio, categorizing data, algorithm training, classifying images, reviewing databases for key information, performing online searches, tagging photos with preset words, and other tasks that are difficult to automate [24].

MTurk has templates for basic surveys that can be customized by the requester via their basic user interface (UI). The UI functions as a low-end word processing software and automatically creates a Hyper Text Markup Language (HTML) based code. The survey was tested in MTurk sandbox, which mimics a live release and allows the writers to see results in real-time without cost. Requesters can take their own survey and see the results using the same interface and server as the live version. After testing in sandbox, we ran a 100 respondent test-run that took 3 hours to complete. This allowed us to tweak any questions that were either unclear or gave ambiguous results.

Workers log in and browse Human Intelligence Tasks (HITs) by title, reward, requester or keyword to find a topic of interest. For our survey, we listed the keywords “answers, survey, experiment, medicine, questionnaire, miscarriage, simple, quick, fun, money, and pregnancy”. When a worker clicks on a requester’s work, the user sees a description of the work prior to accepting the HIT. In the description, we informed workers that they would not be paid for repeating the survey. There is no way to determine the percentage of workers who viewed but did not accept our HIT, but we were able to record how many people accepted our HIT but did not submit.

The system allows requesters to set qualifications for “MTurkers”. Since this was a study regarding the US population, we limited respondents to only those based in the United States. Another qualification we used is the MTurker “HIT approval rate”. The approval rating qualification allows us to select workers who have proven to perform quality work on other tasks. We set the approval rating at 85%, which means that 85% or more of their previous work on various HITs was approved. This is important as it allows us to select higher “quality” workers. We required that workers had finished at least 50 other HITs to increase the likelihood of quality data collection.

HITs are listed in the order they are posted; at any given time there can be thousands of HITs available. We released the surveys in batches of 50. When a worker completes his or her task, the results are automatically sent to the requester for approval and payment. As part of the data received, there is a unique worker ID and an approval rating. The workers’ approval ratings show the percentage of work approved by the requester in the last 7 days, 30 days, and lifetime of the worker. That percentage allows one to filter out anyone who has taken the survey previously and reject his or her work. This method allowed us to control for workers repeating the survey, which would skew the data.

### Data Filter and Cost

We used an attention check question (ACQ) of “Have you had a fatal heart attack while watching TV” as well as a time filter. If the respondent answered yes or maybe to the ACQ or if it took a respondent less than 60 seconds to finish the 23-item survey, they were excluded from the study. Once we finished screening respondents based on the ACQ and time filters, we were able to approve or reject the results. Upon acceptance, respondents were immediately paid US $0.25 for their work. Rejected work was immediately reposted to allow for proper completion. After the survey was completed, the data was downloaded into a Microsoft Excel file for analysis. The study was approved by the Albert Einstein College of medicine institution review board (IRB).

Respondents were aware they were taking a survey regarding pregnancy, however, were unaware of any relationship with an investigator or university. The data was collected over 3 days in January of 2013. The items were not randomized and adaptive questioning was used based on respondents answer to whether they had a miscarriage. If they answered yes to miscarriage, further questions regarding their feelings after a miscarriage were added. The 23-item survey (with additional 10 added if they answered yes to the question regarding miscarriage) appeared on a single HTML page. A completeness check was not required prior to submitting and respondents were able to review their answers prior to submission. We were unable to provide a view rate, as we are unable to determine the number of people who viewed our survey but did not choose to accept. All accepted surveys were completed with a 99% item completeness rate.

All survey data were preserved in the original format for analysis and continuous attributes were compared using *t* tests. All significance values were calculated for 2-sided 95% CIs or *P* values less than .05. Microsoft Excel (2016, Redmond, California) was used for analysis.

## Results

### Recruitment Time

**Figure 1:**
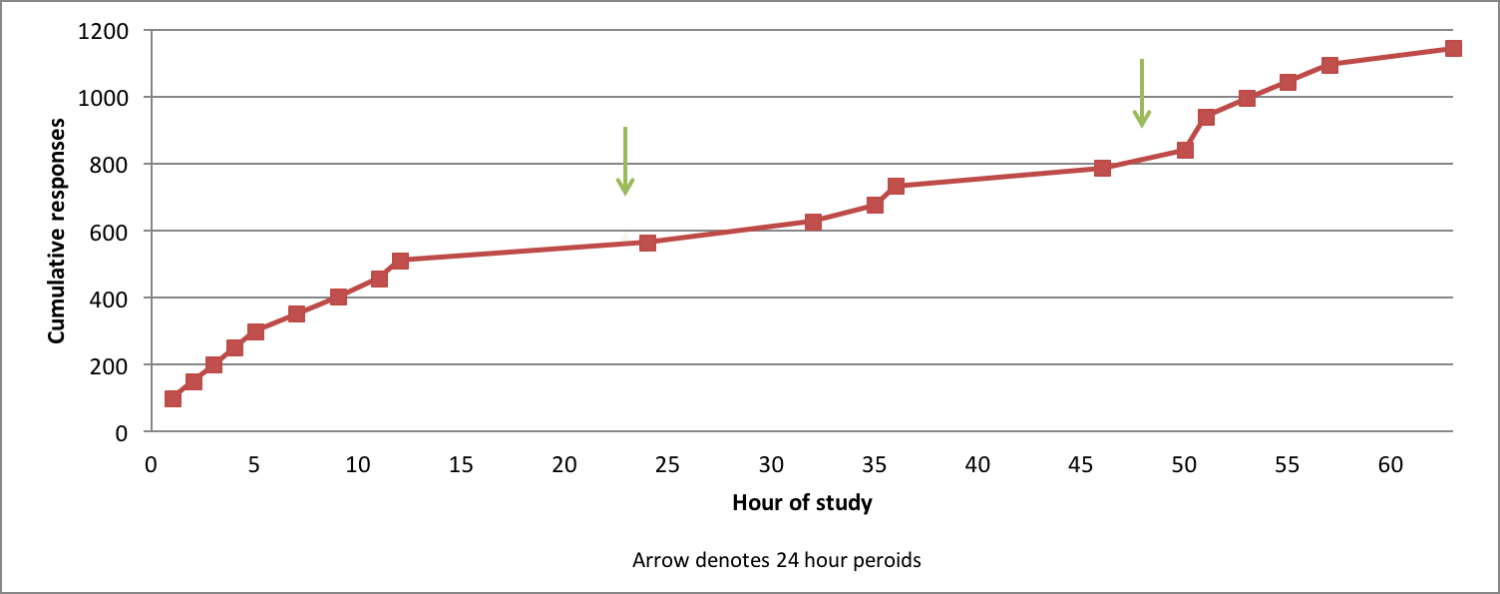
Cumulative responses over time.

A total of 1147 responses were collected in 2 days 14 hours and 42 minutes. On average, this translated to one survey collected every 3.3 minutes. It took 93 minutes to obtain the first 100 responses and 316 minutes to obtain the first 300 responses. The next 845 responses took 57 hours and 26 minutes to collect (Figure 1).

### Data Filter

Of the respondents, 57 repeated the survey, 6 participants answered yes to the ACQ, and no one completed the survey in less than 60 seconds. This left us with a total of 1084 usable responses (usable response rate of 94%).

### Number per Batch

**Figure 2:**
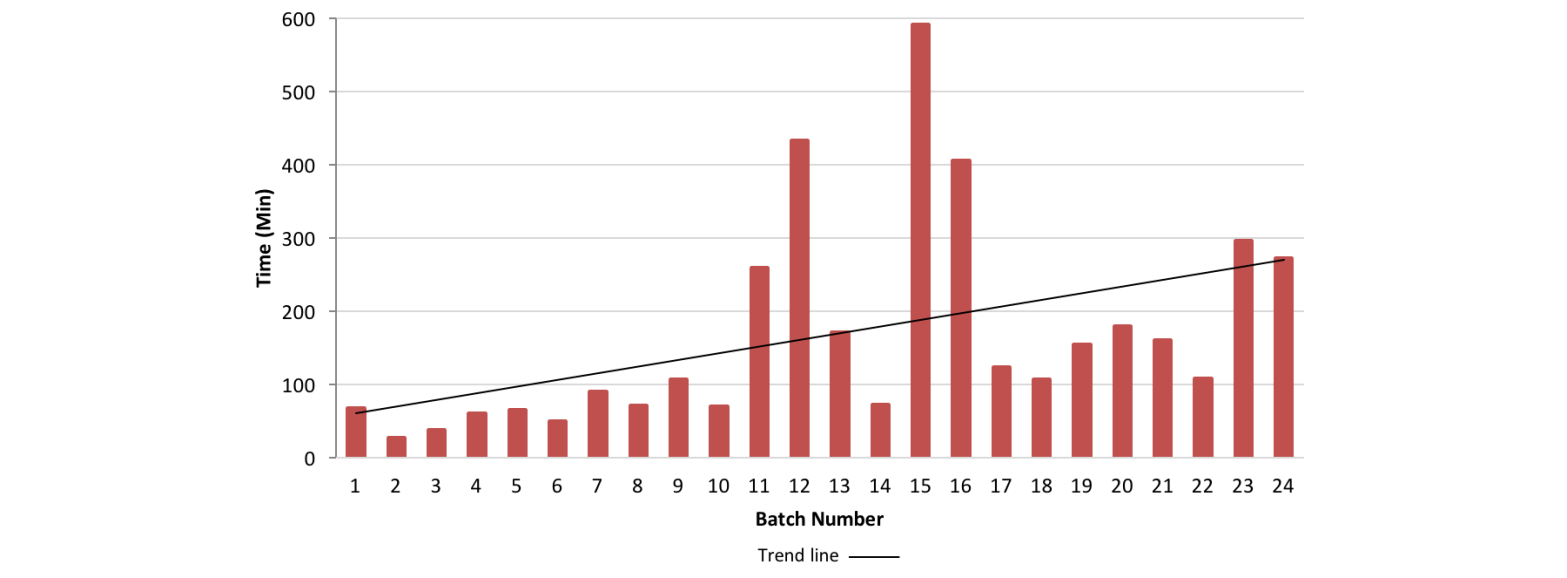
Minutes to batch completion over course of survey.

We found that if we posted batches larger than 50, the batch would take longer to finish and we would cancel the remaining surveys in that particular batch. There were no significant differences in the time to batch completion between posting a batch of 25 (156 min) versus 50 surveys (209 min) (*P*=0.5). Batches posted between 11:00 pm and 8:30 am (Eastern Standard Time, EST) took the longest to complete. With each subsequent batch posted, the average time to completion increased. The shortest time to finish a batch was 26 minutes, and the longest was 591 minutes (Figure 2).

The mean age of the respondents was 31, with the majority of the sample between 18 to 34 years (Table 1). Of the total number participants, 44.74% (485/1084) were male and 54.80% (594/1084) female. In addition, 53.60% (580/1082) were never married, with 37.80% (409/1082) currently married. Most of our respondents were white (82.87%, 895/1080) with 6.29% (68/1080) Hispanic and 5.55% (60/1080) black. Over half the respondents (51.40%, 548/1066) reported a religious affiliation. The majority of religious people identified themselves as Christian (44.93%, 479/1066). The majority of our respondents either attended some college (39.11%, 422/1079) or graduated college (36.79%, 397/1079). As well, 10.19% (110/1079) of the respondents reported graduating high school as their highest degree earned.

The respondents represented a diverse socioeconomic status with 17.14% (17/1079) earning less than US $19,000 a year. The majority of the respondents earned less than US $60,000 (70.06%, 756/1079) with 9.73% (105/1079) earning over US $100,000 a year. We collected responses from 49 of the 50 states. California (9.45%, 102/1079), New York (6.85%, 74/1079), Florida (5.37%, 58/1079), and Pennsylvania (5.28%, 57/1079) were the four largest contributors. Arkansas, Montana and North Dakota had the fewest respondents with each contributing 1 (0.09%, 1/1079).

### Cost

We reimbursed survey respondents US $0.25. We paid a total of US $271.00 to 1084 respondents for completing the survey. The average effective hourly rate for the respondents came out to US $3.97. Since Amazon charges a 10% fee of what is paid to the workers, our total cost for running the survey was US $298.10 or US $0.275 per respondent.

**Table 1:** Participant demographic characteristics (N=1084).

Characteristic		Overall sample^a^, n (%)
Age, years	18-24	342 (31.69)
	25-34	438 (40.59)
	35-44	182 (16.86)
	45-54	70 (6.48)
	over 55	47 (4.35)
Gender		
	Male	485 (44.74)
	Female	594 (54.80)
Marital status	Married	409 (37.80)
	Never married	580 (53.60)
	Divorced	68 (6.28)
	Separated or widowed	25 (2.31)
Race/ethnicity	White	895 (82.87)
	Black	60 (5.55)
	Hispanic^b^	68 (6.29)
	Asian	77 (7.12)
	Other	48 (4.44)
Religion	Christian	479(44.93)
	Judaism	22 (2.06)
	Islam	11 (1.03)
	Buddhism	28 (2.63)
	Other	8 (0.08)
	Any religious affiliation	548 (51.40)
	Unaffiliated (atheist/agnostic)	518 (48.59)
Education	Attended some high school	12 (1.11)
	Graduated high school	110 (10.19)
	Attended Some college	422 (39.11)
	Graduated college	397 (36.79)
	Attended graduate school	128 (11.86)
	Attended medical school	10 (0.09)
Annual income, US dollars	<$19,999	185 (17.14)
	$20,000-39,999	312 (28.91)
	$40,000-59,999	259 (24.00)
	$60,000-79,999	132 (12.23)
	$80,000-99,999	86 (7.97)
	$100,000-249,999	94 (8.71)
	>$250,000	11 (1.01)

^a^Overall sample numbers when added together do not always equal the full sample of 1084 due to missing data points in that category.

^b^In accordance with NIH Racial and Ethnic Categories, Hispanic was a separate question, therefore the total number in this category may add up to greater than 1084.

## Discussion

### Principal Findings

Our study utilized Amazon’s MTurk to effectively and efficiently obtain survey data from a large national pool of both men and women. We were able to obtain both quantitative and qualitative information regarding participants’ knowledge of and experience with miscarriage. What makes this data unique is that we obtained quality data from over 1000 respondents over a 3-day window for under US $300. Obtaining 1000 respondents with other survey methods would cost between 4 to 69 times as much as our method depending on the survey type [4,12]. In addition, it would have taken on average weeks to months to complete the requisite number of responses [5-7].

We found that posting batches larger than 50 led to longer batch completion times, and that the best time to post was between 8:30 am to 8:30 pm EST. Based on our data, researchers should post batches during the hours in which their targeted population is awake. For example, researchers on the east coast who are targeting a west coast population should adjust their posting schedules to match the sleep/wake schedules of their expected participants.

Previously, complex technical computer skills were required to create online surveys [25]. MTurk’s UI is simple to use. For those without technical skills, MTurk can be set to function similar to simple word processing programs. For those with more advanced programming skills, other more powerful programming languages may be added. MTurk, therefore, allows basic and advanced users of computer technology to easily create and distribute surveys.

### Limitations

Limitations to the study include the potential for non-responder bias, as it is not possible to determine how many people previewed our survey without completing it. It is possible that those who responded felt more strongly about issues related to miscarriage. However, these limitations may exist regardless of method of survey distribution.

In addition, previous studies have suggested that the demographics of MTurk participants differed from national demographics; a paper by Paolacci et al suggested that only 47% of MTurk workers were in the United States [18]. However, our study included only those who were US based, thereby eliminating bias from international responders. As prior studies using MTurk have shown [14], race and ethnicity are not proportionately represented, with an underrepresentation of blacks and Hispanics and an overrepresentation of Asians. This may limit the generalizability of our study to the United States as a whole. In addition, respondents had attained a higher level of education than the general public, which may have led to under emphasis of misperceptions found in the general public [26]. However, participants were from 49 of 50 states with no one region over or underrepresented, and overall, the sociodemographic distribution across gender, age, religion, geographic location, and household income was consistent with 2010 national census statistics [27].

### Conclusions

To our knowledge, our study is the first to examine perceptions and understanding of miscarriage among the US national population, and the first to use crowdsourced surveys to examine patient satisfaction with health care providers. Other studies have been able to obtain information from a national sample, however, data collection was costly as well as labor and time intensive. The results of our study demonstrate that MTurk is a safe, cost-effective, and time-efficient way to confidentially obtain important, sensitive information on reproductive health from a large, diverse participant population. Our study also shows that it is also possible to obtain rapid data for general health questions. While many previous studies have assessed MTurk’s validity within psychological and behavioral research, our survey utilized MTurk to conduct medical research, the results of which may impact the future health care of both men and women.

## Appendix

Multimedia Appendix 1 Survey as published online.

## Abbreviations

ACQ: attention check question
EST: Eastern Standard Time
HTML: Hyper Text Markup Language
MTurk: Mechanical Turk
UI: user interface

## References

[1] Aldridge A, Levine K (2001). Surveying the Social World: Principles and Practice in Survey Research.

[2] Blumberg SJ, Ganesh N, Luke JV, Gonzales G (2012). National Health Statistics Report.

[3] Yu J, Cooper H (1983). A quantitative review of research design effects on response rates to questionnaires. J Mark Res.

[4] Sinclair M, O'Toole J, Malawaraarachchi M, Leder K (2012). Comparison of response rates and cost-effectiveness for a community-based survey: postal, internet and telephone modes with generic or personalised recruitment approaches. BMC Med Res Methodol.

[5] Starr K, McPherson G, Forrest M, Cotton SC (2015). SMS text pre-notification and delivery of reminder e-mails to increase response rates to postal questionnaires in the SUSPEND trial: a factorial design, randomised controlled trial. Trials.

[6] Clark L, Ronaldson S, Dyson L, Hewitt C, Torgerson D, Adamson J (2015). Electronic prompts significantly increase response rates to postal questionnaires: a randomized trial within a randomized trial and meta-analysis. J Clin Epidemiol.

[7] Man M, Tilbrook HE, Jayakody S, Hewitt CE, Cox H, Cross B, Torgerson DJ (2011). Electronic reminders did not improve postal questionnaire response rates or response times: a randomized controlled trial. J Clin Epidemiol.

[8] Edwards PJ, Roberts I, Clarke MJ, Diguiseppi C, Wentz R, Kwan I, Cooper R, Felix LM, Pratap S (2009). Methods to increase response to postal and electronic questionnaires. Cochrane Database Syst Rev.

[9] Rea L, Parker R (2014). Designing and Conducting Survey Research: A Comprehensive Guide. Fourth Edition.

[10] Gmel G (2000). The effect of mode of data collection and of non-response on reported alcohol consumption: a split-sample study in Switzerland. Addiction.

[11] Perkins JJ, Sanson-Fisher RW (1998). An examination of self- and telephone-administered modes of administration for the Australian SF-36. J Clin Epidemiol.

[12] Survey Monkey.

[13] (2016). Amazon Mechanical Turk.

[14] Kuang J, Argo L, Stoddard G, Bray BE, Zeng-Treitler Q (2015). Assessing pictograph recognition: a comparison of crowdsourcing and traditional survey approaches. J Med Internet Res.

[15] Buhrmester M, Kwang T, Gosling SD (2011). Amazon's mechanical turk: a new source of inexpensive, yet high-quality, data?. Perspect Psychol Sci.

[16] Horton J, Chilton L (2010). The labor economics of paid crowdsourcing.

[17] Crump MJ, McDonnell JV, Gureckis TM (2013). Evaluating Amazon's Mechanical Turk as a tool for experimental behavioral research. PLoS One.

[18] Paolacci G, Chandler J, Ipeirotis P (2010). Running experiments on Amazon Mechanical Turk. Judgm Decis Mak.

[19] Magid KH, Matlock DD, Thompson JS, McIlvennan CK, Allen LA (2015). The influence of expected risks on decision making for destination therapy left ventricular assist device: an MTurk survey. J Heart Lung Transplant.

[20] Yeh VM, Schnur JB, Margolies L, Montgomery GH (2015). Dense breast tissue notification: impact on women's perceived risk, anxiety, and intentions for future breast cancer screening. J Am Coll Radiol.

[21] Bardos J, Hercz D, Friedenthal J, Missmer SA, Williams Z (2015). A national survey on public perceptions of miscarriage. Obstet Gynecol.

[22] Kong GW, Lok IH, Yiu AK, Hui AS, Lai BP, Chung TK (2013). Clinical and psychological impact after surgical, medical or expectant management of first-trimester miscarriage--a randomised controlled trial. Aust N Z J Obstet Gynaecol.

[23] Musters AM, Koot YE, van den Boogaard NM, Kaaijk E, Macklon NS, van der Veen F, Nieuwkerk PT, Goddijn M (2013). Supportive care for women with recurrent miscarriage: a survey to quantify women's preferences. Hum Reprod.

[24] Mason W, Suri S (2012). Conducting behavioral research on Amazon's Mechanical Turk. Behav Res Methods.

[25] Greenlaw C, Brown-Welty S (2009). A comparison of web-based and paper-based survey methods: testing assumptions of survey mode and response cost. Eval Rev.

[26] (2016). United States Census Bureau.

[27] (2010). United States Census 2010.

