# Supplementary material for: Cloud Based Surveys to Assess Patient Perceptions of Health Care: 1000 Respondents in 3 days for US $300
Source: JMIR Res Protoc. 2016 Aug 23;5(3):e166. doi: 10.2196/resprot.5772 (PMC5013244; doi:10.2196/resprot.5772)
Supplement: Multimedia Appendix 1 [file resprot_v5i3e166_app1.html]

Untitled Document


### Please answer a short survey

This is a voluntary research study about Pregnancy in the United States. All data will remain anonymous and you will receive a token of appreciation through MTurk. In accordance with MTurk policies, your identity will remain unknown and there is no way for researchers to match up your answers with your identity. You may stop answering questions at any time. Upon completion of the survey you will be paid $0.25 (25 cents).

**Background**

 1. What is your current marital status?

Married
Widowed
Divorced
Separated
Never Married

2. Have you or your partner ever been pregnant? (positive pregnancy test)

 Yes   
 No

3. Have you or your partner had a history of a miscarriage? (pregnancy loss earlier than 20 weeks)

Yes   
 No

Was it a planned pregnancy?

 Yes   
 No

Indicate number of miscarriages that occurred:

1. Less than 7 weeks 
(Insert number)  
2. 7-14 weeks 
(Insert number)  
3. >14 weeks 
(Insert number)

Did you access medical care for your miscarriage?

 Yes   
 No

Who did you tell about your loss (check all that apply)?

 Partner  
 Mother  
 Father  
 Sibling  
 Friend  
 Physician/Healthcare Personnel  
 No one  

Please rate the following statements on a scale from 1 to 5 with 1= strongly disagree and 5= strongly agree.

|  |  |  |  |  |  |  |  |
| --- | --- | --- | --- | --- | --- | --- | --- |
|  |  | Strongly Disagree | Disagree | Undecided | Agree | Strongly Agree | Not Applicable |
| Question | **After My Miscarriage...**. |  |  |  |  |  |  |
| A | I received adequate emotional support from those I told | 1 | 2 | 3 | 4 | 5 | N/A |
| B | I received adequate emotional support from my partner | 1 | 2 | 3 | 4 | 5 | N/A |
| C | The medical establishment provided adequate medical support | 1 | 2 | 3 | 4 | 5 | N/A || D | The medical establishment provided adequate emotional support | 1 | 2 | 3 | 4 | 5 | N/A || E | I felt guilty | 1 | 2 | 3 | 4 | 5 |  |
| F | I felt alone | 1 | 2 | 3 | 4 | 5 |  |
| G | I felt ashamed | 1 | 2 | 3 | 4 | 5 |  |
| H | I feel I did something wrong which caused the miscarriage | 1 | 2 | 3 | 4 | 5 |  |
| I | I feel that I could have prevented the miscarriage | 1 | 2 | 3 | 4 | 5 |  |

Was a cause for your miscarriage found?

 Yes   
 No   
 Unsure   
 Did not seek medical care

What reason did medical personnel give you for your miscarriage, if any?

What did you feel was the reason for the miscarriage?

Please rate the following two statements on a scale from 1 to 10 with 1= strongly disagree and 10= strongly agree.

When a  **public figure or celebrity reveals** that they or their spouse had a miscarriage, it makes me feel less alone.

|  |  |  |  |  |  |  |  |  |  |
| --- | --- | --- | --- | --- | --- | --- | --- | --- | --- |
| Strongly Disagree | | Disagree | | Perhaps   Disagree | Perhaps  Agree | Agree | | Strongly Agree | |
| 1 | 2 | 3 | 4 | 5 | 6 | 7 | 8 | 9 | 10 |
|  |  |  |  |  |  |  |  |  |  |

When a **friend of family member** reveals that they or their spouse had a miscarriage, it makes me feel less alone.

|  |  |  |  |  |  |  |  |  |  |
| --- | --- | --- | --- | --- | --- | --- | --- | --- | --- |
| Strongly Disagree | | Disagree | | Perhaps   Disagree | Perhaps  Agree | Agree | | Strongly Agree | |
| 1 | 2 | 3 | 4 | 5 | 6 | 7 | 8 | 9 | 10 |
|  |  |  |  |  |  |  |  |  |  |

4. Has anyone else in your family had a miscarriage?

Yes   
 No   
 I don't know

5. How many biologic children do you have?

0  
 1  
 2  
 3  
 4 or more

6. While watching TV have you ever had a fatal heart attack?

Yes   
 No   
 Maybe

7. In your opinion, what fraction of pregnancies in the United States end in a miscarriage?

|  |
| --- |
| 75% |
| 50% |
| 25% |
| 5% |
| 1% |
| 0.1% |
| 0.01% |

8. What do you think is the  **most common**  cause of miscarriages? (choose only one)

Lifestyle (examples include: drugs, alcohol, smoking during the pregnancy)  

Genetic (examples include: age of mother, genetic problems with fetus)  

Medical Problems (examples include: hormonal, uterine)  

Psychological Issues ( examples include: stressful event, depression, mother not wanting the pregnancy)  
 Punishment from God  
 Destiny or Fate

9. Please select if you agree or disagree with the following statements**: The following can be a cause of miscarriage:**

|  |  |  |  |  |
| --- | --- | --- | --- | --- |
|  |  | Agree | Disagree | Unsure |
| Question | **The Following can be a cause of miscarriage...**. |  |  |  |
| A | Punishment from God | Agree | Disagree | Unsure |
| B | Getting into an argument | Agree | Disagree | Unsure |
| C | Lifting heavy objects | Agree | Disagree | Unsure |
| D | Woman not wanting the pregnancy | Agree | Disagree | Unsure |
| E | Premarital sex | Agree | Disagree | Unsure |
| F | Sexual intercourse during pregnancy | Agree | Disagree | Unsure |
| G | Past use of birth control | Agree | Disagree | Unsure |
| H | Jealousy | Agree | Disagree | Unsure |
| I | Longstanding stress | Agree | Disagree | Unsure |
| J | A stressful event | Agree | Disagree | Unsure |
| K | Genetic abnormalities of the fetus | Agree | Disagree | Unsure |
| L | Moderate exercise (equivalent of 20 minutes on the treadmill) | Agree | Disagree | Unsure |
| M | Having had a sexually transmitted disease in the past | Agree | Disagree | Unsure |
| N | Having had an abortion in the past | Agree | Disagree | Unsure |
| O | Past use of an IUD ( Intrauterine Device) | Agree | Disagree | Unsure |
| P | Spiritual causes such as destiny or fate | Agree | Disagree | Unsure |

10. What would a miscarriage mean for you emotionally? On a scale from 1-5 with 1= extremely upsetting, 5=not upsetting.

|  |  |  |  |  |
| --- | --- | --- | --- | --- |
| Extremely upsetting:   Like the loss of a child |  | Moderately upsetting |  | Not upsetting:   only an inconvenience |
| 1 | 2 | 3 | 4 | 5 |
|  |  |  |  |  |

11. Would you want to **know the cause** of the miscarriage if there **was** something you could do to prevent the miscarriage from happening in the future? On a scale from 1-5 scale with 1= strongly not like to know and 5= strongly like to know.

|  |  |  |  |  |
| --- | --- | --- | --- | --- |
| Strongly not like to know | Would not like to know | Unsure | Would like to know | Strongly like to know |
| 1 | 2 | 3 | 4 | 5 |
|  |  |  |  |  |

12. Would you want to **know the cause** of the miscarriage even if there was **NOT** something you could do to prevent the miscarriage from happening in the future? On a scale from 1-5 scale with 1= strongly not like to know and 5= strongly like to know.

|  |  |  |  |  |
| --- | --- | --- | --- | --- |
| Strongly not like to know | Would not like to know | Unsure | Would like to know | Strongly like to know |
| 1 | 2 | 3 | 4 | 5 |
|  |  |  |  |  |

13. What is your gender?

Male   
 Female

 14. In what year were you born?

 15a. Are you Hispanic/Latino?

Yes   
 No

 15b. What is Your Race or Ethnic Group?

White
Black, African Am. or Negro
American Indian or Alaska Native
Chinese
Japanese
Korean
Vietnamese
Other Asian
Caribbean
Pacific Islander
Other

 16. Which religion do you identify with? Scroll down to view all options

Christianity- Catholic
Christianity- Protestant
Christianity- Orthodox
Christianity - Mormon

Christianity - Other 
Judaism- Orthodox
Judaism- Conservative
Judaism- Reform
Judaism- Other
Islam
Unaffiliated (Atheist, Agnostic)
Buddhism
Hinduism

If you chose other please specify here:

17. On a Scale from 1-5, How important is Religion in your life? 1= very unimportant and 5 = very important

|  |  |  |  |  |
| --- | --- | --- | --- | --- |
| Very unimportant | Unimportant | Neutral/Unsure | Important | Very Important |
| 1 | 2 | 3 | 4 | 5 |
|  |  |  |  |  |

 18. Which of the following best describes your highest achieved education level? Scroll down for more options.

Attended Elementary School
Attended Some High School
Graduated High School
Attended Some College
Graduated College
Attended Graduate School
Attended Medical School

 19.What is the total income of your household before taxes? Scroll down for more options.

<$19,999
$20,000-39,999
$40,000-59,999
$60,000-79,999
$80,000-99,999
$100,000-249,999
>$250,000

 20. What is your Political Affiliation?

|  |  |
| --- | --- |
|  | Democrat |
|  | Republican |
|  | Independent |
|  | None |

21. What is your political persuasion? On a 1-5 scale with 1= very liberal, 5=very conservative

|  |  |  |  |  |
| --- | --- | --- | --- | --- |
| Very liberal | Liberal | Neutral/Unsure | Conservative | Very conservative |
| 1 | 2 | 3 | 4 | 5 |
|  |  |  |  |  |

22. What is your position on abortion?

|  |  |  |  |
| --- | --- | --- | --- |
| There should be   no law limiting abortion | There should be   some limits to abortion | Abortion should be   illegal in all circumstances | Unsure |
|  |  |  |  |

 23. Which state do you live in?

Alabama
Alaska
Arizona
Arkansas
California
Colorado
Connecticut
Delaware
District of Columbia
Florida
Georgia
Hawaii
Idaho
Illinois
Indiana
Iowa
Kansas
Kentucky
Louisiana
Maine
Maryland
Massachusetts
Michigan
Minnesota
Mississippi
Missouri
Montana
Nebraska
Nevada
New Hampshire
New Jersey
New Mexico
New York
North Carolina
North Dakota
Ohio
Oklahoma
Oregon
Pennsylvania
Rhode Island
South Carolina
South Dakota
Tennessee
Texas
Utah
Vermont
Virginia
Washington
West Virginia
Wisconsin
Wyoming

Thank you for completing this survey.
